# Alignment accuracy and functional outcomes between hand-held navigation and conventional instruments in TKA: a randomized controlled trial

**DOI:** 10.1186/s12891-022-05872-y

**Published:** 2022-11-26

**Authors:** Rapeepat Narkbunnam, Chaturong Pornrattanamaneewong, Pakpoom Ruangsomboon, Keerati Chareancholvanich

**Affiliations:** Division of Adult Reconstructive Surgery, Department of Orthopaedic Surgery, Faculty of Medicine Siriraj Hospital, Mahidol University, 2 Wanglang Road, Bangkoknoi, 10700 Bangkok, Thailand

**Keywords:** Hand-held accelerometer-based navigation, iAssist, Total knee arthroplasty, Alignment accuracy, Alignment, Functional outcomes

## Abstract

**Background:**

This study assessed surgical accuracy and functional outcomes using hand-held accelerometer-based navigation following total knee arthroplasty (TKA). Question: (1) Does hand-held navigation (the iAssist system) improve surgical accuracy (assessed with five parameters commonly used to evaluate surgical alignment: the hip-knee-ankle angle (HKA), femoral coronal angle (FCA), tibial coronal angle (TCA), femoral sagittal angle (FSA), and tibial slope angle (TSA)) compared to conventional instruments in TKA? (2) Which surgical alignment parameters among HKA, FCA, TCA, FSA, and TSA can obtain the most advantage from the iAssist system? (3) Does the iAssist system lead to better functional outcomes at two years of follow-up after TKA?

**Methods:**

This parallel-group double-blinded randomized controlled trial recruited 60 patients (30 patients each in the iAssist and conventional group) with osteoarthritis who underwent primary TKA by a single surgeon at Siriraj Hospital. There was no loss to follow-up in the study. All procedures in both groups were performed using similar surgical exposure, prosthesis implant, perioperative and postoperative protocols. Participants in the iAssist group received the iAssist system as an assistive technique, while those in the conventional group only had conventional instruments. Surgical alignments (HKA, FCA, TCA, FSA, and TSA) were recorded using CT scan at six weeks post-operation. Functional outcomes were assessed with knee ROM, KSS, and EQ-5D at 6 months, 1 year and 2 years post-operation. Baseline characteristics including age, sex, the affected knee side, and body mass index were comparable between the two groups, similar to preoperative ROM, KSS, and EQ-5D.

**Results:**

The mean operative time was relatively longer in the iAssist than the conventional group, although not statistically significant (88.1 ± 13.7 versus 83.4 ± 21.3; *p* = 0.314). Among the surgical alignment parameters evaluated, FCA was the only radiographic parameter with a statistically significant difference between the two groups and was closer to 90º in the iAssist group (89.4 ± 2.2 in the iAssist versus 87.2 ± 2.1 in the conventional group; *p* = 0.003). Also, there was a higher proportion of outliers in the conventional than the iAssist group (23.3% versus 10%; *p* = 0.086). Nonetheless, HKA and TCA did not differ between the two groups (*p* = 0.25 and 0.096, respectively), although the percentages of outliers were higher in the conventional than the iAssist group (HKA: 26.7% vs. 13.3%; *p* = 0.101 and TCA: 6.7% versus 0%; *p* = 0.078). Likewise, we observed other radiographic parameters had no significant group differences, including FSA and TSA. Furthermore, at two years post-operation, we found no differences between the iAssist and the conventional group in knee ROM (106.7 ± 14.6 versus 108.2 ± 12.7; *p* = 0.324), KSS (82.5 ± 6.4 versus 83.8 ± 3.4; *p* = 0.324), and EQ-5D (0.9 ± 0.2 versus 1.0 ± 0.1; p = 0.217). All functional outcomes were also comparable at 6 months and 12 months of follow-up postoperatively.

**Conclusion:**

The iAssist technology increase surgical precision by allowing for a more precise FCA with fewer outliers than conventional equipment. iAssist had longer operative time. Functional outcomes and quality of life were not different.

**Level of evidence:**

I

## Introduction

Restoring physical function and improving mobility are the most important objectives of undergoing total knee arthroplasty (TKA). Although the procedure has been widely performed and has shown excellent long-term survivorship, yet there remain factors that may cause aseptic loosening and instability leading to unfavorable outcomes [1]. The alignment of the limb is one of the major determinants of functional outcomes and prosthesis survivorship [2, 3]. Previous studies have agreed that a mechanical alignment that exceeds 3˚ of varus or valgus is considered a malalignment [4, 5].

Innovative technologies help surgeons improve surgical technique and prosthesis function. They benefit surgeons in preoperative, intraoperative, and postoperative decision-making. [6, 7]. These technologies have been shown to contribute to a more accurate alignment of the lower limb and better orientation precision of prosthetic components [1, 8, 9]. Comparing assistive technologies with conventional instruments, however, has not shown a substantial improvement in the functional result of patients. [9, 10]. Moreover, they consume more healthcare resources, most of which were at higher costs, thereby causing a financial burden. Also, they generally require a longer operative time [6, 11, 12]. Consequently, the benefits of implementing these navigation technologies have still been controversial.

The iAssist Knee system (Zimmer, Warsaw, IN, USA), hand-held accelerometer-based navigation, was introduced as a combination of computer-assisted surgery (CAS) and conventional instruments. However, unlike CAS, the iAssist system does not mandate substantial administrative burden or excessive costs [13, 14]. Hand-held navigation has helped surgeons position prostheses more accurately, improve lower limb mechanical alignments, and reduce outliers [15–17]. Its accuracy is not well-established due to a lack of data utilizing computed tomography (CT scan), the most reliable tool for postoperative radiographic outcome evaluation. Few research have compared hand-held accelerometer-based navigation to conventional instruments. [18–21]. Also, no randomized controlled trial has been conducted to evaluate its functional outcomes at at least two years of follow-up. Therefore, we conducted this randomized controlled trial to determine surgical accuracy assessed with postoperative CT scan and compare functional outcomes at two years post-operation between the iAssist system and conventional instruments in TKA.

Our research questions included (1) Does hand-held navigation (the iAssist system) improve surgical accuracy (assessed with five parameters commonly used to evaluate surgical alignment: the hip-knee-ankle angle (HKA), femoral coronal angle (FCA), tibial coronal angle (TCA), femoral sagittal angle (FSA), and tibial slope angle (TSA)) compared to conventional instruments in TKA? (2) Which surgical alignment parameters among HKA, FCA, TCA, FSA, and TSA can obtain the most advantage from the iAssist system? (3) Does the iAssist system lead to better functional outcomes at two years of follow-up after TKA?

## Materials and methods

This parallel-group double-blinded randomized controlled trial had recruited patients who underwent primary TKA between November 2016 and May 2018 at Siriraj Hospital, Mahidol University, Thailand. Included participants were randomized in a 1:1 ratio to either the iAssist or the conventional group. The randomization sequence was computer-generated and concealed using sealed opaque envelopes. After preoperative randomization and treatment allocation, the iAssist group received the surgery performed with the assistance of the hand-held navigation system. Whilst no assistive device was employed in the conventional group. All participants were blinded to their randomized treatment. There are no additional patient payments. Participants were not charged any additional fees. This study was prospectively registered in the ClinicalTrials.gov (NCT03111407). Full date of first registration was on 12/04/2017. All patients provided written informed consent prior to their participation in the study. There was no protocol deviation after the study had commenced.

The inclusion criteria were adult patients with osteoarthritis who underwent unilateral primary TKA. Patients with at least one of the following conditions were excluded: inflammatory arthritis, previous septic arthritis, traumatic osteoarthritis, preexisting extra-articular deformities, severe knee deformities (varus or valgus deformity greater than 15°), flexion contracture greater than 10°, complex TKA due to significant bone loss, preexisting comorbidities (American Society of Anesthesiologists (ASA) physical status classification more than or equal to 3). All participants were followed for a minimum of two years.

### Variables, Radiographic Assessment, Clinical Outcome Measures

**Fig. 1:**
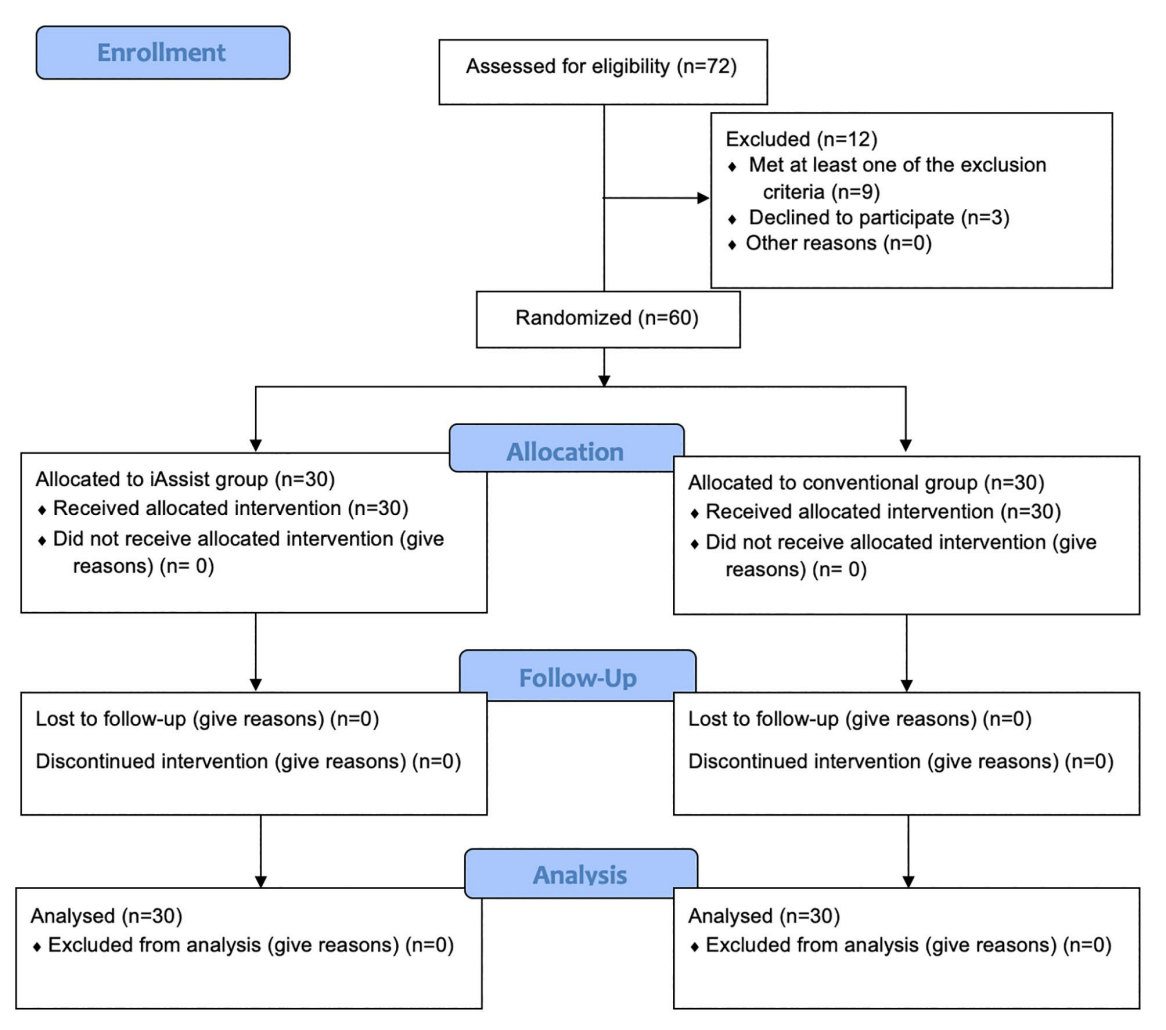
The figure presents the consolidated standards of reporting trials (CONSORT) flow diagram of participants in the study.

To evaluate and compare alignment accuracy between the two interventions, we determined parameters of mechanical alignments by using a lower-limb CT scan performed at six weeks after TKA. Five parameters (HKA, FCA, TCA, FSA, and TSA) were estimated by two surgeons independently and blinded to both the treatment arm of each patient and the measurements of the other assessor. The mean value of the two measurements was recorded for each parameter. HKA is an angle between the femur’s mechanical axis and that of the tibia, which is generally aimed at 180˚ (Fig. 1 A). For coronal alignment measurements, the CT slides were rotated until the best true AP view of the prosthesis, where medial and lateral femoral condyles are symmetrical, can be identified. In this view, we assessed FCA, an angle lied between the mechanical axis of the femur and the transcondylar line of the femoral prosthesis (Fig. 1B), and TCA, an angle between the mechanical axis of the tibia and the base of the tibial prosthesis (Fig. 1 C), both angles expected to be 90˚. To analyze sagittal alignments, the CT slides were rotated until the best true lateral view of the prosthesis, where there is no overlapping between the medial and lateral condyles, is achieved. FSA is an angle between the distal femoral prosthesis line and the line drawn from the center of the femoral head to the midpoint of the femoral prosthesis (Fig. 1D), which is aimed at 3˚ flexion. While TSA is the angle between the tibial base plate line and the line drawn from the midpoint of the tibial prosthesis to that of the tibial plafond. (Fig. 1E). For all of the angles evaluated, malalignment was defined as at least 3 degrees higher or lower than the angles aimed. Accordingly, we also analyzed the percentage of outliers (the implants that aligned out of ± 3º from the target angles) for each of the angles assessed. Furthermore, we evaluated functional outcomes with knee range of motion (ROM), Knee Society Scores (KSS), KSS functional scores, health-related quality of life assessment using EQ-5D platform, and EQ-5D: health state at 6 months, 1 year, and 2 years postoperatively.

Demographic and clinical data of each patient was collected prior to the surgery, including age, gender, the affected knee side, BMI, and functional outcomes. Preoperative ROM, KSS, KSS functional score, EQ-5D, and Eq. 5D: health state were comparable between the two study groups (Table 1).

**Table 1:** Physical performances and quality of life compared between the iAssist and the conventional group

Outcome measure	iAssist (*n* = 30)				Conventional (*n* = 30)				P value for Comparisons at 2 years
	**Preop**	**6 mo**	**1 yr**	**2 yr**	**Preop**	**6 mo**	**1 yr**	**2 yr**	
Range of Motion	106.0 ± 16.4	111.2 ± 10.4	117.1 ± 9.8	106.7 ± 14.6	109.5 ± 13.6	117.7 ± 10.2	119.2 ± 8.4	108.2 ± 12.7	0.324
Knee Society Scores	65.8 ± 9.7	80.5 ± 8.3	82.3 ± 6.5	82.5 ± 6.4	68.4 ± 12.0	82.9 ± 4.3	83.7 ± 3.4	83.8 ± 3.4	0.324
Knee Society Function Scores	71.5 ± 15.4	76.0 ± 12.8	71.5 ± 15.4	71.5 ± 15.4	76.0 ± 12.8	71.5 ± 15.4	76.0 ± 12.8	76.0 ± 12.8	0.223
EQ-5D	0.6 ± 0.2	0.8 ± 0.2	0.9 ± 0.2	0.9 ± 0.2	0.6 ± 0.2	0.9 ± 0.1	0.9 ± 0.1	1.0 ± 0.1	0.217
EQ-5D: Health State	65.9 ± 15.4	80.3 ± 9.6	80.5 ± 10.4	78.8 ± 12.6	63.6 ± 19.9	81.7 ± 8.9	81.3 ± 8.5	82.8 ± 7.3	0.138

Notes: data presented as mean ± standard deviation

### Surgical procedures

All procedures in both groups were performed using the medial parapatellar approach by a single surgeon. A conventional intramedullary alignment guide was used to make a distal femoral cut, which was set at 6º of valgus. Whilst a proximal tibia cut was made by using an extramedullary alignment guide. The cut is expected to be perpendicular to the tibial mechanical axis resulting in 3º of the posterior tibial slope. In the iAssist group, the iAssist system was employed to assist the surgeon in achieving the alignment setting. In this study, all patients received the same prosthesis design, NexGen LPS-Flex (Zimmer Inc., Warsaw, IN, USA), a cemented, posterior-stabilized, fixed-bearing knee prosthesis. No patella was resurfaced. Extensor mechanisms were repaired. Also, wound closure was operated as per our regular routine. All patients were immediately allowed for full-weight bearing after the surgery. The same postoperative in-hospital rehabilitation protocol provided by the hospital’s physical therapist team was applied to all participants. Early ROM exercises and progressive ambulation with supporting devices were encouraged for all patients. After hospital discharge, patients were advised to continue a standard home-based exercise program without an out-patient visit for rehabilitation. No postoperative complications were encountered, and no patients required reoperation in the study.

### Sample size calculation and statistical analysis

Descriptive statistics were employed, and their results are presented as mean ± SD for continuous or frequency and percentage for categorical variables. We performed between-group comparisons of continuous data with unpaired t-test because all variables were normally distributed based on the Kolmogorov-Smirnov test. While qualitative variables were compared using the Chi-squared test. SPSS Statistics (SPSS, Inc., Chicago, IL, USA) was obtained for all statistical analyses. A *p* value less than 0.05 was considered statistically significant.

We calculated the sample size based on the means and SD of HKA and FCA from Kawaguchi et al.’s study [19]. With 5% type I error, approximately 20 and 30 participants per group were required to achieve 80% power for HKA and FCA, respectively. Therefore, we enrolled 30 participants per group (60 in total) in order to answer both the study outcomes with adequate statistical power.

## Results

**Fig. 2A-E:**
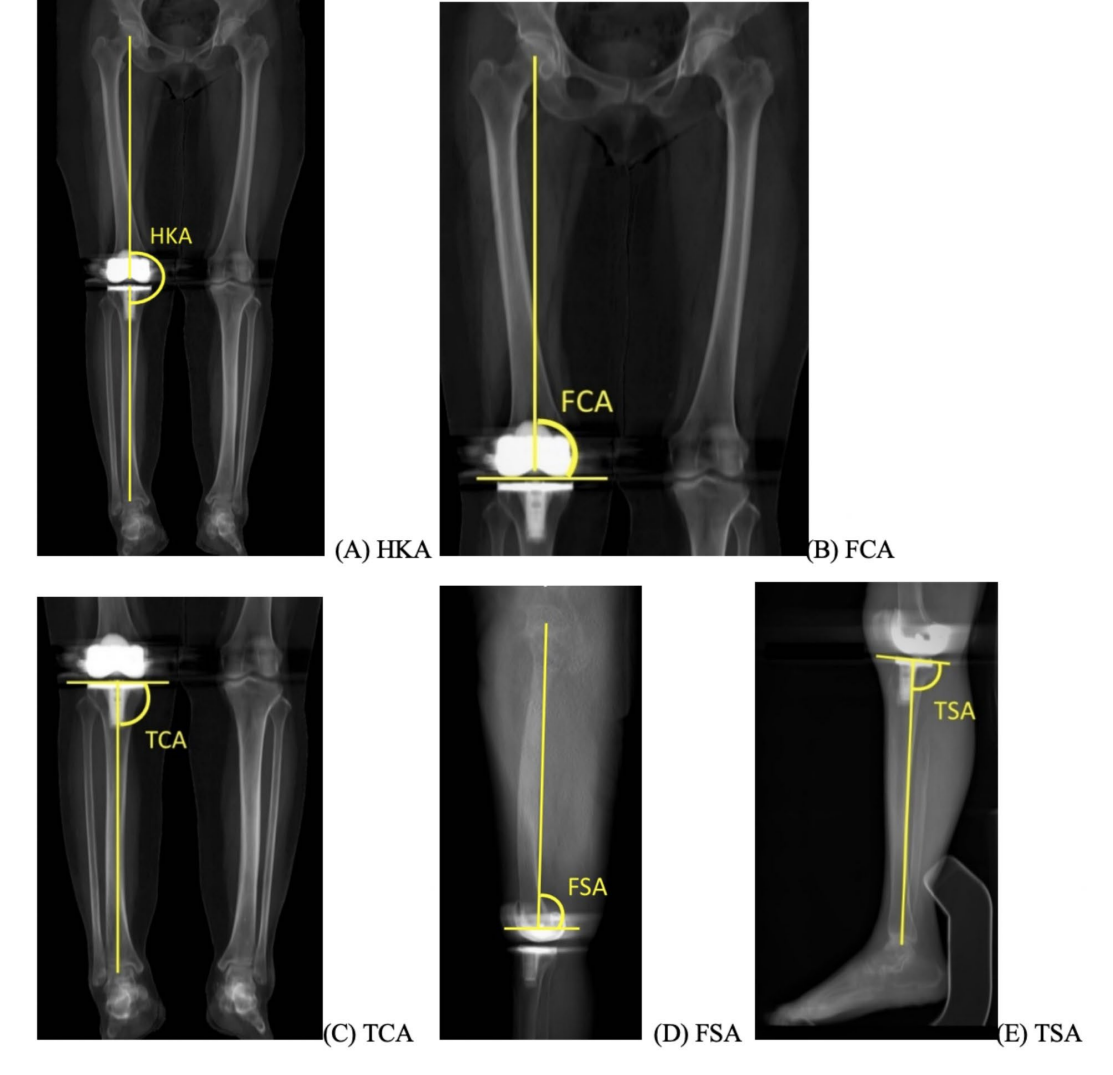
These photos depict radiographic measurements with CT scans in the coronal plane to evaluate HKA (A), FCA (B), and TCA (C), and in the sagittal plane for FSA (D), and TSA (E)

A total of 72 patients were assessed for eligibility. Of these, 12 patients were excluded (8 met at least one of the exclusion criteria and 3 declined to participate). Consequently, 60 patients were enrolled and randomized; 30 in each group. No patients were lost to follow-up (Fig. 2). In the iAssist group, there were 27 females and 3 males with a mean age of 67.5 (standard deviation; SD 7.6) years. The conventional group also consisted of 27 females and 3 males, and they had an average age of 66.4 (SD 6.3) years. The affected side of the knee and body mass index (BMI) were similar between the two study groups (p = 0.795 and 0.510, respectively), as shown in Table 2. Operative time was longer in the iAssist than in the conventional group (88.1 ± 13.7 versus 38.4 ± 21.3); however, the difference did not meet statistical significance (p = 0.314).

**Table 2:** Patient demographic and clinical characteristics compared between the iAssist and the conventional group

Patient Characteristics	iAssist (***n*** = 30)	Conventional (***n*** = 30)	***P*** value
Age, year	67.5 ± 7.6	66.4 ± 6.3	0.547
Female	27 (90.0%)	27 (90.0%)	1.000
Right affected knee side	14 (46.7%)	13 (43.3%)	0.795
Body mass index, kg/m^2^	28.2 ± 5.8	27.3 ± 4.4	0.510
Operation time, min	88.1 ± 13.7	83.4 ± 21.3	0.314

Notes: data presented as mean ± standard deviation for continuous and frequency (%) for categorical data

As presented in Table 3, at six weeks post-operation, FCA was closer to 90º for iAssist patients than for those who only got conventional devices (89.4 ± 2.2 versus 87.7 ± 2.1, *p* = 0.003). Although HKA and TCA in the iAssist group were also closer to the expected angles (HKA 179.7 ± 2.2 versus 178.9 ± 2.9 and TCA 90.1 ± 1.1 versus 90.7 ± 1.5), the differences were not statistically significant (*p* = 0.250 and 0.096, respectively). Similarly, no significant difference was observed in FSA even though the mean angle was more misaligned in the iAssist group (3.8 ± 2.0 versus 3.2 ± 0.9; *p* = 0.267). Additionally, TSA and tibial slopes were similar between the two groups (tibial slope 3.8 ± 2.3 and 3.6 ± 1.9 in the iAssist and the conventional group, respectively; p = 0.668). Knee society score, knee society functional score, EQ-5D, and EQ-5D Health State did not demonstrate any statistically significant differences across all time points (Table 1).

**Table 3:** Mechanical alignments measured at 6 weeks post-operation compared between the iAssist and the conventional group

Mechanical alignment	iAssist (***n*** = 30)	Conventional (***n*** = 30)	***P*** value
Hip-knee-ankle angle, degree	179.7 ± 2.2	178.9 ± 2.9	0.250
Hip-knee-ankle angle outlier	4 (13.3%)	8 (26.8%)	0.101
Femoral coronal alignment, degree	89.4 ± 2.2	87.7 ± 2.1	0.003
Femoral coronal alignment outliner	3 (10%)	7 (23.3%)	0.086
Tibial coronal alignment, degree	90.1 ± 1.1	90.7 ± 1.5	0.096
Tibial coronal alignment outlier	0 (0%)	2 (6.7%)	0.078
Femoral sagittal alignment, degree^a^	3.8 ± 2.0	3.2 ± 1.9	0.267
Femoral sagittal alignment outlier	3 (10%)	2 (6.7%)	0.321
Tibial slope, degree^b^	3.8 ± 2.3	3.6 ± 1.9	0.668
Tibial slope outlier	2 (6.7%)	2 (6.7%)	0.5

Notes: data presented as mean ± standard deviation for continuous and frequency (%) for categorical data

^a^Femoral sagittal alignment = 90 – FSA (+ component flexion, - component extension)

^b^Tibial slope = 90 – TSA (+ posterior slope, - anterior slope)

Most of the time, the iAssist group had fewer outliers than the conventional group.; these differences were found in HKA (13.3% versus 26.7%), FCA (10% versus 23.3%), and TCA (0% versus 6.7%), although they did not meet statistical significance (p = 0.101, 0.086, and 0.078, respectively). Additionally, there were only minor to no differences in the percentages of outliers in FSA and tibial slope (FSA 10% versus 6.7% in the iAssist and conventional group; tibial slope 6.7% in both groups).

## Discussion

In this study, we sought to determine if implementing the iAssist system in addition to conventional instruments could provide more accurate lower limb alignments compared to conventional technique alone; if yes, which angle could earn the most benefits and if these benefits also lead to better functional outcomes.

Several previous studies have shown that the hand-held accelerometer-based navigation system could optimize the accuracy of the surgical alignments compared to conventional instruments [14–21]. Two recent systematic reviews reported favorable results of HKA, FCA, and TCA in favor of the innovative technique; however, only small effect sizes and inconclusive results were observed in FSA [22, 23]. Thiengwittayaporn et al. found fewer outliers of HKA, FCA, and TCA in the hand-held navigation group [15]. While Seow-Hng Goh et al. failed to demonstrate statistically significant differences regarding mechanical axes and their outliers, coronal femoral-component angle, and coronal tibial-component angle when compared between patients receiving the accelerometer-based navigation, computer-assisted surgery, and conventional instruments [18].

The results of the present study were in the same direction as these previous studies and systematic reviews. We observed that FCA was the only radiographic measurement with a statistically significant difference between the iAssist and conventional group, with the latter having a higher proportion of outliers. At the same time, other radiographic measurements were similar between the groups. The reason behind our results might have been because of the incidence of excess femoral bowing, one of the important factors affecting the accuracy of FCA, especially under conventional intramedullary guides [24, 25]. This incidence rate is known to be high in the Asian population [26]. Since the main advantage of the hand-held navigation system is the derivation of more accurate alignments not deviated by femoral canal geometry, this innovative technology can help surgeons to accurately locate the center of hip rotation, while standard instruments are employed under intramedullary guides to make the distal femoral cut. In contrast, extramedullary instruments are applied for proximal tibial resection at the same landmarks used to evaluate the tibial alignments whether or not the hand-held navigation instrument is employed. Therefore, these mechanisms could have explained why FCA, unlike tibial alignments, was the only significant parameter. In fact, we believe that the accuracy of the tibial alignments depends predominantly on the surgeon’s experience rather than the navigation instruments used for assistance. Nevertheless, compared with previous studies, the present study yielded more accurate precision since the measurements were made using CT scans instead of plain radiography. With CT scans, one can adjust the rotational planes of the femoral and tibial prosthesis until the best true AP and lateral views are derived upon measuring the alignments. This represents an advantage of CT scan over scanogram, in which such fine-tuning at the time of measurement is not possible.

In previous studies, the iAssist system have failed to deliver better physical performances and quality of life compared to the conventional technique [18–21]. Unfortunately, most of these studies evaluated only short-term outcomes, and only a few studies have reported these outcomes at or after two years post-operation. Seow-Hng Goh et al. found that ROM, KSS, Oxford Knee Score, and SF-36 at six months and two years postoperatively were similar between the study groups [18]. The most recent retrospective study by Gao et al. compared 24 patients who had the hand-held accelerometer-based navigation assistance with 274 propensity-score-matched patients who received conventional instruments. Similarly, they found no significant differences in physical performances (ROM and KSS) and quality of life (EQ-5D) at 0.5, 1, and 2 years of follow-up [21]. However, the present study delivered concordant results to these previous studies, showing no significant improvement in functional outcomes, including health-related quality of life, in favor of the accelerometer-based navigation system as has been hypothesized. This could have been partly due to the relatively short follow-up duration, as has been discussed previously.

Furthermore, we discovered that implementing the iAssist system resulted in a slightly and insignificantly longer operative time, which was concordant with previous studies and systematic reviews [27, 28]). Another advantage of the hand-held navigation system in comparison to other technology-assisted interventions is that it requires less operative time than other innovative technologies. Systematic reviews have shown that the mean operative durations of both the computer-assisted and robotic-assisted surgery were significantly longer than the conventional technique (mean difference 32 and 21.5 min, respectively) [27, 28]. However, as discovered in the present study, the hand-held navigation system did not need the same amount of time to operate because an extra-articular pin is attached with the computer tracker, and the registration process is fast and simple.

## Limitations

This study has some notable limitations. Firstly, the sample size was relatively small. We were unable to perform a subgroup analysis assessing the effect of femoral bowing on the accuracy of the femoral coronal plane. In fact, many insignificant differences in the present study, such as the percentages of outliers, could have been partly due to the small number of participants and hence the low proportion of outliers. Had there been more participants included, the differences might have been statistically significant. Therefore, the results of this study may be too preliminary to suggest clinical decision-making. Secondly, two years of follow-up might have been too short a duration to demonstrate clinical outcomes of the intervention in preventing instability or aseptic loosening. Accordingly, future studies with a larger number of participants assessing longer-term clinical outcomes are mandatory before making a definite conclusion regarding the benefits of computer-enhanced accelerometer-based navigation in improving functional outcomes after TKA.

## Conclusion

The iAssist technology increase surgical precision by allowing for a more precise FCA with fewer outliers than conventional equipment. iAssist had longer operative time. Functional outcomes and quality of life were not different.

## Data Availability

The datasets generated and/or analyzed during the current study are not publicly available. These datasets were stored in our internal high-security level hard drive but are available from the corresponding author on reasonable request. Requests for data not shown in the body of this manuscript can be made to the corresponding author.

## Declarations

### Trial registration

The protocol was reviewed and approved by Siriraj Institutional Review Board [238/2557(EC3)] and prospectively registered in the ClinicalTrials.gov (NCT03111407). Full date of first registration was on 12/04/2017.

## Abbreviations

**HKA**: Hip knee ankle angle
**FCA**: Femoral coronal angle
**TCA**: Tibial coronal angle
**FSA**: Femoral sagittal angle
**TSA**: Tibial slope angle
